# Ferroptosis in the tumor microenvironment: mechanisms, advances, and therapeutic perspectives

**DOI:** 10.3389/fonc.2025.1650219

**Published:** 2025-08-22

**Authors:** Weijuan Gao, Jiani Tan, Chengtao Yu

**Affiliations:** The First Clinical School of Nanjing University of Chinese Medicine, Nanjing, China

**Keywords:** ferroptosis, tumor microenvironment, lipid peroxides, iron, antioxidants

## Abstract

Ferroptosis is a regulated, non-apoptotic form of cell death marked by the accumulation of iron-dependent lipid peroxides. This process causes rapid rupture of the plasma membrane and the release of intracellular contents. Ferroptosis acts as an intrinsic tumor-suppressive mechanism. It plays a crucial role in tumor progression, metastasis, and resistance to standard therapies, including chemotherapy and radiotherapy. Its unique molecular mechanisms confer significant therapeutic potential. In recent years, multiple experimental therapies aiming to induce ferroptosis have been developed for cancer treatment. Although these therapies show promise in controlling tumor growth, their effects on the tumor microenvironment (TME) require further investigation. Recent studies indicate that distinct cell populations within the TME have different sensitivities to ferroptosis. This variability may lead to unintended effects, such as damage to normal cells or increased inflammation, resulting in toxicity. Cells in the TME can either undergo ferroptosis or modulate its regulation through intercellular signaling and interactions. Notably, ferroptosis-related interactions between tumor cells and other components of the TME, such as immune cells, stromal cells, and endothelial cells, are central to TME remodeling. This mini-review summarizes recent advances in ferroptosis mechanisms and highlights the dynamic interplay between ferroptosis and the TME. It also discusses the prospects and challenges of ferroptosis-based cancer therapies.

## 1 Introduction

While organismal death marks the end of life, death at the cellular level is essential for maintaining tissue homeostasis and the overall survival of multicellular organisms. Based on triggering factors, cellular context, and morphological features, cell death can be broadly classified into accidental cell death (ACD) and regulated cell death (RCD) (1). Unlike ACD, which is passive and often results from acute cellular damage, RCD is an active and tightly regulated process that contributes to growth, development, and tissue homeostasis. A classic example of RCD is the orderly elimination of interdigital cells during fetal development, which is critical for proper limb formation (2). Based on distinct molecular mechanisms, RCD can be further divided into several types, including autophagy-dependent cell death, apoptosis, necroptosis, pyroptosis, and ferroptosis (3).

Ferroptosis is a distinct form of RCD with unique mechanisms and significant potential for cancer therapy. Compared to pyroptosis, which is mediated by gasdermin proteins and triggers strong inflammatory and immune responses, ferroptosis offers a more selective therapeutic approach. While pyroptosis can amplify anti-tumor immunity, its lack of specificity often causes tissue damage and immune-related side effects because it is activated in both tumor and normal cells (4, 5). In contrast, ferroptosis is a caspase-independent process driven by iron accumulation, lipid peroxidation, and inhibition of the system Xc-/GSH/GPX4 pathway. These features enable ferroptosis to exploit the iron dependency of tumor cells, potentially inducing cell death with greater selectivity and minimizing effects on normal tissues. Furthermore, unlike apoptosis, which is often evaded by tumor cells through mutations in key regulators such as p53, ferroptosis bypasses these resistance mechanisms. This makes it particularly effective against therapy-resistant cancers. By regulating iron metabolism and oxidative stress, ferroptosis can enhance tumor cell sensitivity to treatment. It also holds promise for improving the tumor immune microenvironment when combined with immunotherapy (6–8).

Resistance to RCD is a hallmark of cancer. In particular, resistance to apoptosis allows tumor cells to maintain unlimited proliferative capacity. Targeting non-apoptotic cell death mechanisms has emerged as a rational and promising therapeutic strategy in cancer treatment (9). Iron is a critical element required for cellular proliferation and metabolism. Many tumor cells exhibit iron overload, likely driven by their increased demand for rapid growth. However, iron acts as a “double-edged sword” for tumor cells. While iron overload meets the metabolic needs of rapid proliferation, excessive iron can disrupt cellular iron homeostasis and trigger ferroptosis (10, 11). The term “ferroptosis” was first introduced in 2012 by the laboratory of Brent R. Stockwell. During ferroptosis, cells show characteristic morphological changes, such as cell shrinkage, loss of plasma membrane integrity, and chromatin condensation (12). Inducing ferroptosis is considered an effective strategy to overcome tumor cell resistance to apoptosis. Notably, combining ferroptosis induction with therapies such as immunotherapy or radiotherapy can significantly enhance tumor-killing efficacy (13). However, studies have also identified instability and potential risks associated with ferroptosis-based treatments. A major concern is the toxic effects on non-tumor cells within the tumor microenvironment (TME), which may reduce therapeutic efficacy and increase treatment-related risks (14).

The tumor microenvironment is a dynamic and heterogeneous ecosystem that includes tumor cells, immune cells, stromal cells, and extracellular matrix components. These elements play key roles in tumor progression, immune evasion, and therapy resistance (15). Ferroptosis and the TME are closely connected through bidirectional interactions. Ferroptosis can modulate immune responses and cellular behavior, and it is also regulated by signals within the TME (16). Moreover, immune cells show different sensitivities to ferroptosis. Different immune cell types can either promote or inhibit ferroptosis in tumor cells, further altering the dynamic balance of the TME. This interplay not only reshapes the TME but also profoundly influences tumor progression and therapeutic strategies (16, 17). This review discusses the molecular mechanisms of ferroptosis, its bidirectional interactions with the TME, and potential therapeutic strategies for cancer treatment.

## 2 Ferroptotic mechanisms of action

**Figure 1:**
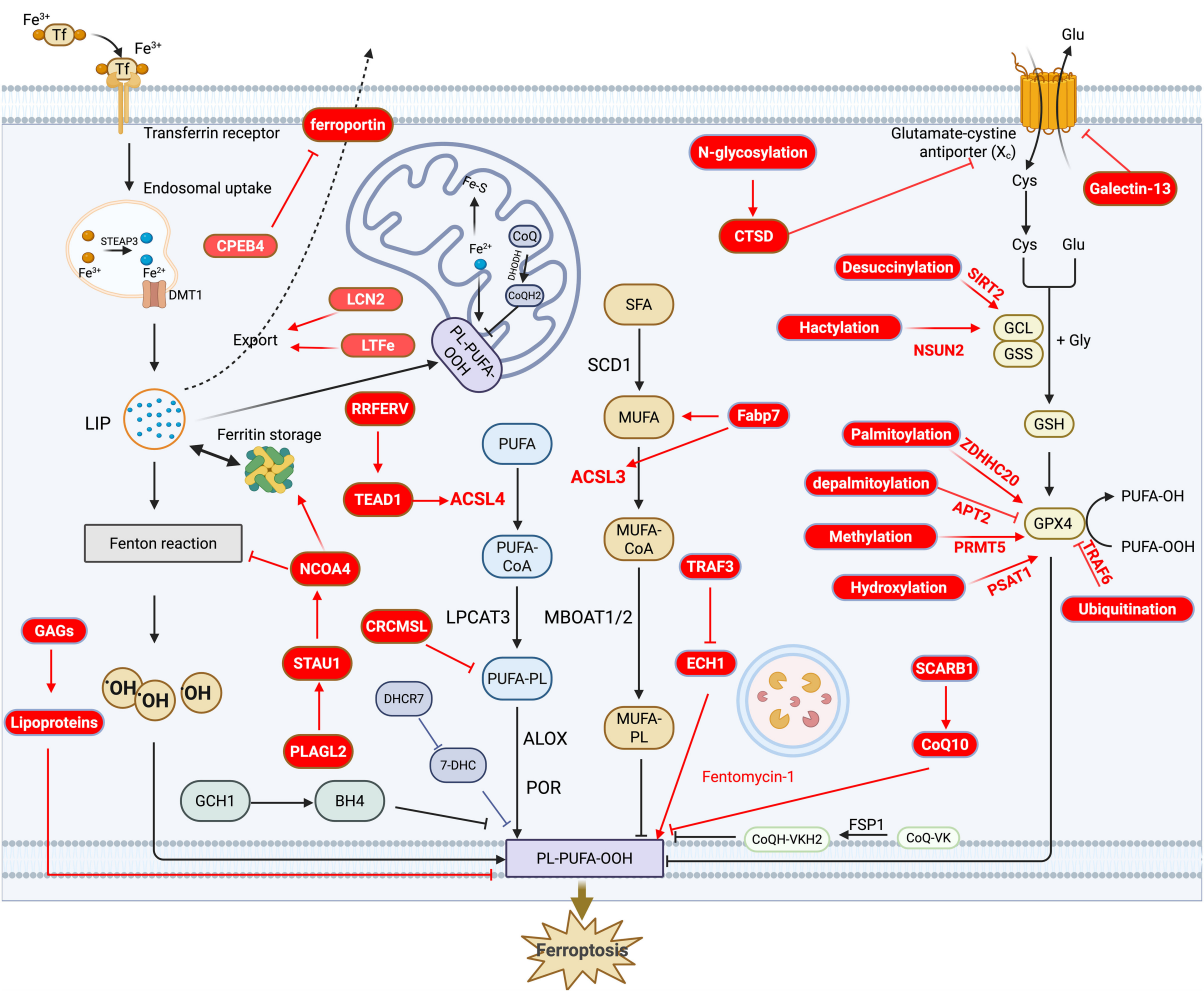
Overview and recent advances in the molecular mechanism of ferroptosis. This diagram highlights the three major mechanisms of ferroptosis: the lipid oxidation pathway, the iron metabolism pathway, and the redox pathway. Recent advancements are indicated in red, while other colors provide an overview of ferroptosis. Specifically, in the lipid oxidation pathway, recent progress focuses on the regulation of genes such as Fabp7, ACSL3, ACSL4, CRCMSL, and RRFERV, which play key roles in ferroptosis. In the iron metabolism pathway, newly identified regulators, including CPEB4, LCN2, LTFe, and NCOA4, have been shown to influence ferroptosis by modulating iron metabolism. For the redox pathway, the latest advances center on post-translational modifications of GPX4, such as palmitoylation, depalmitoylation, and methylation, which are critical for ferroptosis regulation.

Ferroptosis is characterized by iron-dependent lipid peroxidation and is primarily associated with lipid metabolism, iron metabolism, redox systems, and the involvement of various organelles. In this section, we first provide a systematic overview of the molecular mechanisms underlying ferroptosis, followed by a summary of the latest advancements in this field. The mechanistic diagram is presented in **Figure 1**. For more detailed insights into the molecular mechanisms, readers are referred to the cited references (18–21).

### 2.1 Overview of molecular mechanisms

#### 2.1.1 Lipid peroxidation

The terminal event of ferroptosis is plasma membrane rupture driven by the accumulation of specific lipid peroxides, particularly phospholipid hydroperoxides (PLOOH), a process that depends on the incorporation and oxidation of polyunsaturated fatty acids (PUFAs) (22). Lipid peroxidation occurs through both enzymatic and non-enzymatic processes, each involving distinct pathways and regulatory mechanisms. In the enzymatic pathway, lipoxygenases (LOXs) and cytochrome P450 oxidoreductase (POR) act as key regulators by catalyzing the oxidation of PUFAs and facilitating the formation of lipid hydroperoxides. LOXs catalyze the formation of PLOOH from esterified PUFAs, which accumulate within the plasma membrane, leading to structural instability and subsequent peroxidation (23). POR facilitates electron transfer to oxygen, generating hydrogen peroxide, which acts as a reactive oxygen species (ROS) to initiate and amplify lipid peroxidation (24). In the non-enzymatic pathway, acyl-CoA synthetase long-chain family member 4 (ACSL4) and lysophosphatidylcholine acyltransferase 3 (LPCAT3) are representative regulators. Both facilitate ferroptosis by activating PUFAs and incorporating them into membrane phospholipids (25–27). Additionally, emerging evidence suggests that sterol metabolism can indirectly regulate ferroptosis by modulating phospholipid remodeling and levels of reactive trapping agents (28).

Recent advances have identified 7-dehydrocholesterol (7-DHC), a key intermediate in the distal cholesterol biosynthesis pathway, as a potent endogenous suppressor of ferroptosis. Mechanistically, 7-DHC possesses an exceptionally high reactivity toward peroxyl radicals, enabling it to outcompete polyunsaturated fatty acid (PUFA) phospholipids for radical trapping. By preferentially undergoing oxidation, 7-DHC diverts lipid peroxidation away from membrane phospholipids, thereby protecting cellular membranes from peroxidation-mediated damage and blocking ferroptosis execution. Both genetic deficiency and pharmacological inhibition of DHCR7 (the enzyme that converts 7-DHC to cholesterol) lead to 7-DHC accumulation, conferring robust resistance to ferroptosis in cancer cells and promoting more aggressive tumor phenotypes *in vivo*. Conversely, inhibition of 7-DHC synthesis sensitizes cancer cells to ferroptosis and suppresses tumor growth. These findings establish the 7-DHC–DHCR7 axis as a critical checkpoint in ferroptosis regulation and highlight its therapeutic potential as a target for enhancing ferroptosis in cancer therapy or suppressing ferroptosis in acute tissue injury (29, 30).

#### 2.1.2 Iron metabolism

As mentioned previously, peroxidation of membrane-bound lipids enriched in PUFAs is a hallmark of ferroptosis, and this process is primarily driven by the Fenton reaction. The Fenton reaction is mediated by ferrous iron (Fe²^+^) within the labile iron pool (LIP), generating highly reactive hydroxyl radicals that induce lipid peroxidation. The size of the LIP is determined by iron metabolism, including iron absorption, storage, transport, and export. Specifically, the iron–transferrin complex enters cells via transferrin receptor 1 (TFR1)-mediated endocytosis, forming endosomes in which ferric iron (Fe³^+^) is reduced to Fe²^+^ by six-transmembrane epithelial antigen of prostate 3 (STEAP3), and then transported into the LIP by divalent metal transporter 1 (DMT1). Intracellular iron can be exported via ferroportin (FPN1) (31). However, dysregulated iron metabolism, which leads to elevated intracellular iron levels, not only enhances the activity of enzymes such as lipoxygenases (LOXs), thereby promoting lipid peroxidation, but also expands the LIP, facilitating the Fenton reaction and hydroxyl radical production, ultimately triggering ferroptosis (32, 33).

#### 2.1.3 Redox system

The redox system maintains cellular homeostasis by regulating the production and clearance of reactive oxygen species (ROS), thereby protecting cells from oxidative damage. During ferroptosis, both glutathione peroxidase 4 (GPX4)-dependent and GPX4-independent antioxidant pathways contribute to maintaining redox balance. GPX4 directly reduces PLOOH to the corresponding PLOH, and its activity depends on glutathione (GSH), which is synthesized from cysteine transported by the xCT system (34). GPX4-independent pathways mainly involve the reactive trapping antioxidant system, including FSP1/coenzyme Q (CoQH2), DHODH/CoQH2, and GCH1/BH4. CoQ and its reduced form, CoQH2, inhibit ferroptosis by scavenging lipid peroxyl radicals. FSP1 inhibits plasma membrane lipid peroxidation by reducing CoQ10 to CoQH2 and consuming NAD(P)H, and also functions as a vitamin K reductase with antioxidant properties (35). DHODH detoxifies lipid peroxides in mitochondria via the reduction of CoQ10, while GCH1 inhibits ferroptosis through both BH4 synthesis and the regulation of lipid remodeling (36).

#### 2.1.4 Organelles

Mitochondria play a dual role in regulating ferroptosis sensitivity. Anti-ferroptotic functions include MFN1-mediated mitochondrial fusion, which reduces ferroptosis sensitivity in cancer cells, as well as the activation of stress response pathways via DELE1 and ATF4, which enhance GSH synthesis and GPX4 stability (37). Moreover, mitochondria are involved in the synthesis of the anti-ferroptotic molecule CoQ, and limit lipid peroxidation by degrading PUFAs via enzymes such as DECR1 (38, 39). Pro-ferroptotic functions involve the tricarboxylic acid cycle and glutaminolysis, which promote ferroptosis by increasing oxidative stress (40). Increased mitochondrial iron uptake and disruption of Fe–S cluster biogenesis further enhance ferroptosis sensitivity, potentially related to cystine deprivation (20, 41).

The endoplasmic reticulum (ER) also plays a central role in ferroptosis by regulating lipid metabolism, ROS generation, and transcription factor processing. ER-resident enzymes such as POR and CYB5R1 generate ROS using NADPH, thereby initiating membrane lipid peroxidation (42). The ER is a hub for lipid metabolism, with enzymes such as FADS1 and FADS2 orchestrating the balance between PUFA and monounsaturated fatty acid (MUFA) metabolism, thereby determining ferroptosis sensitivity (43, 44). Other organelles, including lipid droplets and peroxisomes, also participate in the regulation of ferroptosis. Further details are available in the cited literature (20, 45, 46).

### 2.2 Recent progression of molecular mechanisms in cancer

The research on ferroptosis has progressed rapidly, particularly in the field of cancer, with critical regulatory factors being continuously identified. Here, we explore the latest advancements from three perspectives: lipid peroxidation, iron metabolism and redox systems.

#### 2.2.1 Updates in lipid peroxidation

Ferroptosis sensitivity is tightly regulated by lipid metabolism in cancer cells, with distinct mechanisms across different cancer types. In *in vivo* CRISPR screening experiments, ACSL4 was identified as a key enzyme promoting metastasis through its role in lipid metabolism. Mechanistic studies have demonstrated that ACSL4 enhances metastasis by increasing the fluidity of the plasma membrane, thereby promoting tumor cell migration and invasion. Interestingly, the increased membrane fluidity induced by ACSL4 simultaneously enhances the sensitivity of metastatic tumor cells to ferroptosis, highlighting its dual role as both a metastasis promoter and a ferroptosis sensitizer. Notably, the combination of ACSL4 inhibition and suppression of fatty acid β-oxidation has been shown to more effectively hinder ovarian cancer cell metastasis. These findings suggest that the increased membrane fluidity of metastatic tumor cells may underlie their heightened sensitivity to ferroptosis (47). In breast cancer, dormant disseminated tumor cells (DTCs) are characterized by elevated *de novo* lipogenesis and overexpression of ACSL3. Suppression of lipogenesis and ACSL3 expression in dormant breast cancer cells shifts the lipid profile from being rich in MUFA to one enriched in PUFA. This shift results in lipid peroxide accumulation, ultimately triggering ferroptosis. These findings suggest that dormant cells may evade ferroptosis as a survival mechanism, and targeting ferroptosis could represent a promising strategy to prevent tumor recurrence (48). These studies highlight ACSL enzymes as crucial regulators of lipid metabolism in both metastatic and recurrence progression.

Fatty acid-binding proteins (FABPs), such as FABP7, play a critical role in fatty acid metabolism and transport by binding long-chain fatty acids. In murine lung cancer cell lines, FABP7 enables tumor cells to evade immune cell-induced ferroptosis. Mechanistically, FABP7 regulates epigenetic reprogramming, downregulates LPCAT3, and upregulates BMAL1 to suppress ferroptosis. Moreover, tumor cells can induce FABP7 expression in CD8+ T cells, leading to T cell apoptosis and facilitating tumor immune escape (49). TNF receptor-associated factors (TRAFs) are a family of intracellular signal transduction adaptors that interact with multiple receptors, including TNFR, TLR, and IL receptors, and play a central role in innate immune signaling. TRAF3, a key member of this family, is frequently suppressed in glioblastoma (GBM) due to promoter hypermethylation. Restoration of TRAF3 expression sensitizes GBM cells to ferroptosis, potentially through TRAF3-mediated K63-linked ubiquitination of enoyl-CoA hydratase 1 (ECH1). This modification prevents the mitochondrial translocation of ECH1, thereby promoting polyunsaturated fatty acid (PUFA) oxidation and lipid peroxidation (50).

Scavenger receptor type B1 (SR-B1) is a multiligand membrane receptor protein and a physiologically important high-density lipoprotein (HDL) receptor. Its primary function is to mediate the selective uptake of HDL-derived cholesterol esters by cells. In a whole-genome CRISPR-Cas9 screening experiment, SCARB1 was identified as a negative regulator of ferroptosis. Mechanistic studies revealed that SCARB1 overexpression simultaneously increases cholesterol and coenzyme Q10 levels-two downstream metabolites in the lipid biosynthesis pathway-to inhibit ferroptosis (51). Tumor cells sustain rapid growth by activating the fatty acid *de novo* biosynthesis pathway; however, some tumor cells can also enhance their utilization of circulating lipids by absorbing low-density lipoproteins (LDL) and high-density lipoproteins (HDL). A CRISPR/Cas9-based functional genomic screening experiment revealed that cancer cells take up lipoproteins through a pathway dependent on sulfated glycosaminoglycans (GAGs) attached to cell surface proteoglycans. Disruption of GAG biosynthesis or acute degradation of surface GAGs significantly reduced lipoprotein uptake, sensitized cancer cells to ferroptosis, and inhibited tumor growth in mice. This may be due, in part, to the fact that lipoproteins carry at least five lipids with anti-ferroptosis activity. Vitamin E, vitamin K2, and coenzyme Q10 function as free radical-trapping antioxidants, while phospholipids containing monounsaturated fatty acids (MUFAs), such as oleic acid, decrease membrane susceptibility to peroxidation (52).

Interestingly, non-coding RNAs also emerge as key players in ferroptosis regulation. For example, lncRNA-RRFERV functions as a competing endogenous RNA (ceRNA) by sponging miR-615-5p and miR-1293. This interaction stabilizes TEAD1 mRNA expression, thereby promoting the malignant progression and radioresistance of nasopharyngeal carcinoma. High expression of RRFERV also regulates the ACSL4/TFRC axis, maintaining an exquisitely balanced redox state in tumor cells, which renders them particularly sensitive to ferroptosis (53). In contrast, CRCMSL promotes ferroptosis by disrupting phospholipid unsaturation and reducing membrane fluidity, adding complexity to the regulatory network (54).

#### 2.2.2 Updates in Iron metabolism

In sarcoma and pancreatic ductal adenocarcinoma, lysosomal iron activation via fentomycin-1 induces ferroptosis by degrading phospholipids, effectively targeting metastatic and drug-tolerant cells. However, sublethal exposure of fentomycin-1 promotes ferroptosis resistance through mesenchymal marker downregulation and membrane repair (55). Similarly, gastric cancer resists ferroptosis via the PLAGL2-STAU1-NCOA4 axis, where STAU1 destabilizes NCOA4 mRNA, suppressing ferritinophagy and reactive iron accumulation (56). Conversely, mechanical stress enhances ferroptosis sensitivity by activating the NCOA4-FTH1 pathway, thereby establishing a mechanobiological link to ferroptosis regulation (57).

Iron homeostasis is another critical factor. In liver cancer, CPEB4 deficiency reduces ferroptosis by suppressing hepcidin and increasing ferroportin expression, while in aged lung cells, the NUPR1-lipocalin-2 axis induces functional iron insufficiency, limiting stemness and tumorigenesis but promoting ferroptosis resistance (58, 59).

Epigenetic regulation also modulates ferroptosis. In prostate cancer, LTFe enhances ferroptosis by promoting lactotransferrin-mediated iron transport, a process disrupted by androgen receptor (AR) signaling. Co-targeting AR and ferroptosis pathways suppresses tumor growth, offering therapeutic potential (60).

#### 2.2.3 Updates in redox system

Redox systems have been extensively studied, with recent research shedding light on the role of post-translational protein modifications (PTMs) in ferroptosis regulation. Post-translational modifications are critical in controlling GPX4 stability. For instance, GPX4 palmitoylation by ZDHHC20 enhances its stability, while APT2-mediated depalmitoylation promotes ferroptosis in colorectal cancer and metastasis models (61). Similarly, PRMT5-mediated arginine methylation stabilizes GPX4 by preventing ubiquitination. Inhibition of PRMT5 sensitizes tumors to ferroptosis, thereby enhancing therapeutic efficacy (62). In addition, GPX4 hydroxylation by PSAT1, activated by IFNγ in triple-negative breast cancer (TNBC), suppresses ferroptosis and reduces immunotherapy efficacy, whereas blocking this pathway restores ferroptosis sensitivity and enhances PD-1 antibody response (63).

Other studies highlight GPX4-targeted degradation. GPX4-AUTAC, exploiting TRAF6-mediated ubiquitination and p62-driven autophagy, selectively degrades GPX4, inducing ferroptosis in breast cancer, especially in combination with chemotherapy (64). Beyond GPX4, cancer cells utilize mechanisms such as SIRT2-regulated desuccinylation of GCLC to boost glutathione synthesis, conferring ferroptosis resistance under oxidative stress (65). Similarly, NSUN2 lactylation in gastric cancer enhances GCLC-dependent glutathione synthesis, promoting ferroptosis resistance in acidic tumor environments (66).

Metastasis-related mechanisms also modulate ferroptosis. In colorectal cancer, N-glycosylation of CTSD by DDOST and STT3B suppresses ferroptosis by regulating ACSL4 and SLC7A11, facilitating liver metastasis (67). In contrast, Galectin-13 secreted by ferroptotic cells promotes ferroptosis propagation by inhibiting SLC7A11 membrane localization in neighboring cells, enhancing tumor sensitivity to ferroptosis inducers and immunotherapy (68).

## 3 The role of ferroptosis in tumor microenvironment

### 3.1 Overview of ferroptosis in tumor microenvironment

Ferroptotic cancer cells exert dual roles within the TME by releasing a variety of signals, which can both activate antitumor immune responses and promote the formation of an immunosuppressive TME. Damage-associated molecular patterns (DAMPs) such as HMGB1, ATP, oxidized phospholipids, and calreticulin from ferroptotic cells stimulate dendritic cell maturation, macrophage phagocytosis, and CD8^+^ T cell infiltration, and upregulate MHC-I and ULBPs, thereby enhancing immune responses. However, under certain conditions, DAMPs and metabolites like 8-OHG and prostaglandin E2 can drive immunosuppression by promoting M2 macrophage polarization, recruiting MDSCs, increasing PD-L1 expression, and suppressing the functions of T cells, NK cells, DCs, and activating Tregs, collectively contributing to an immunosuppressive TME (13, 69).

Immune cells play a key regulatory role in the ferroptosis of tumor cells. CD8^+^ T cells and CAR-NK cells promote ferroptosis by secreting IFN-γ, which downregulates system Xc- and upregulates ACSL4, enhancing lipid peroxidation and inhibiting tumor growth. Conversely, tumor-associated macrophages and adipocytes suppress ferroptosis through TGF-β1, miR-660-5p, and oleic acid secretion, promoting tumor progression. Antitumor immune cells appear to promote tumor cell ferroptosis, while immunosuppressive cells and adipocytes inhibit it, collectively influencing tumor fate (17, 69).

Ferroptosis also impacts tumor-infiltrating immune cells, thereby shaping the immune microenvironment. Induced ferroptosis impairs the antitumor functions of CD8^+^ T cells, NK cells, and dendritic cells, while inhibition restores their activity. In macrophages, ferroptosis favors the immunosuppressive M2 phenotype; its inhibition promotes tumoricidal M1 polarization. GPX4 inhibition induces Treg ferroptosis, reducing immunosuppression but risking autoimmunity. Ferroptosis in MDSCs may also contribute to tumor progression (17, 70).

### 3.2 The progress of ferroptosis in tumor microenvironment

#### 3.2.1 Updates in tumor cells

Recent studies have uncovered various mechanisms through which ferroptosis influences tumor immunity and cancer progression. In melanoma, zDHHC8 suppresses ferroptosis by palmitoylating GPX4 at cysteine 75 (Cys75). Inhibiting zDHHC8 with the small-molecule compound PF-670462 reduces GPX4 palmitoylation, promotes ferroptosis, and enhances CD8^+^ T cell infiltration, thereby suppressing tumor growth (71). Similarly, in non-small cell lung cancer (NSCLC), downregulation of BIN1 inhibits ferroptosis via the G3BP1/STAT1/GSH pathway, which impairs CD8^+^ T cell function and promotes immune evasion (72).

In addition, combining the BCL-2 inhibitor sonrotoclax with radiotherapy enhances anti-tumor immune responses through multiple mechanisms. This combination downregulates GPX4, induces immunogenic ferroptosis, and triggers the release of damage-associated molecular patterns (DAMPs), which activate the NF-κB pathway in tumor-associated macrophages. Meanwhile, cytoplasmic DNA accumulation activates the cGAS–STING pathway, promoting type I interferon release and CD8^+^ T cell activation. This treatment also upregulates PD-L1 expression, and further combination with anti-PD-L1 therapy significantly improves therapeutic efficacy (73).

#### 3.2.2 Updates in T cells

Ferroptosis plays a critical role in CD8^+^ T cell-mediated antitumor immunity, with both intrinsic and extrinsic mechanisms regulating T cell function. PCIF1, an m6A methyltransferase, suppresses CD8^+^ T cell activation by inhibiting ferroptosis suppressor genes such as Fth1 and Slc3a2 via m6A modification, thereby promoting ferroptosis and reducing antitumor immunity. Knockout of PCIF1 increases tumor-infiltrating CD8^+^ T cell numbers and ferroptosis resistance, leading to improved antitumor immunity and enhanced responses to anti-PD-1 therapy and CAR-T cell treatment. Clinically, low PCIF1 expression is associated with better immunotherapy outcomes, suggesting that PCIF1 could be a potential therapeutic target to enhance T cell function (74). Sickle cell disease (SCD) induces ferroptosis in CD8^+^ T cells by disrupting 3D genome architecture, downregulating anti-ferroptotic genes such as SLC7A11, and impairing hydrogen sulfide (H_2_S) biogenesis, which is essential for maintaining redox homeostasis. Restoring H_2_S mitigates ferroptosis and improves CD8^+^ T cell-mediated immunity in SCD models, highlighting the interplay between genetic disorders and tumor immunity (75). In NSCLC, combining ROR1 CAR-T cells with ferroptosis inducers enhances tumor cell ferroptosis by increasing IFN-γ secretion, lipid peroxidation, and ACSL4 upregulation, thereby promoting robust antitumor responses (76). Additionally, disulfidptosis, a distinct cell death pathway, contributes to CD8^+^ T cell exhaustion. LDHB drives disulfidptosis by limiting NADPH availability, depleting glucose-6-phosphate dehydrogenase activity, and promoting STAT3-mediated T cell exhaustion. Targeting LDHB prevents CD8^+^ T cell dysfunction and enhances antitumor immunity, offering new opportunities for therapeutic intervention (77).

#### 3.2.3 Updates in macrophage

Macrophage ferroptosis can promote inflammation and tissue damage. In abdominal aortic aneurysm (AAA), SENP3 increases macrophage ferroptosis and aggravates inflammation. When SENP3 is deficient, ferroptosis in macrophages is reduced. This leads to less inflammatory signaling and fewer macrophages in the aortic wall, which slows AAA development in mouse models. SENP3 promotes ferroptosis by de-SUMOylating cystathionine γ-lyase (CTH), an enzyme important for H_2_S production. Interventions that target the SENP3/CTH pathway or provide H_2_S donors, such as ATB346, may be effective for AAA treatment (78). In colorectal cancer, macrophage resistance to ferroptosis is mediated by Gsta4, which protects against Enterococcus faecalis-induced ferroptosis. Inactivation of Gsta4 triggers ferroptosis by increasing heme oxygenase 1 (Hmox1), phosphorylated c-Jun, and intracellular iron levels, while suppressing GPX4 activity. The resulting ferroptosis eliminates macrophages, ultimately disrupting the microbiota-induced bystander effect and thereby inhibiting colitis and colorectal cancer progression (79).

#### 3.2.4 Updates in myeloid-derived suppressor cells

TIPE2 regulates ferroptosis susceptibility in myeloid-derived suppressor cells (MDSCs) by reprogramming the composition of phosphatidylethanolamine and phosphatidylcholine involved in lipid peroxidation. TIPE2-deficient MDSCs resist IKE-induced ferroptosis by upregulating SLC7A11 and GPX4, reducing lipid ROS, alleviating immunosuppression, and promoting T cell proliferation and infiltration. Blocking TIPE2 in MDSCs enhances the efficacy of combined ferroptosis induction and anti-PD-L1 therapy, providing a novel strategy for liver cancer treatment (80).

#### 3.2.5 Updates in neutrophil

Ferroptosis occurs in neutrophils themselves, particularly in chemoresistant neutrophils with reduced MBOAT1 expression. These ferroptotic neutrophils release immunosuppressive factors, including prostaglandin E2, indoleamine 2,3-dioxygenase, and oxidized lipids, thereby suppressing CD8^+^ T cell activity. This immunosuppressive process is regulated by IL1β^+^CXCL3^+^CD4^+^ T cells via the IL1β/IL1R1/NF-κB signaling pathway, which also promotes replenishment of the neutrophil pool. Targeting Fer-CD4^+^ T cells or IL1R1^+^ neutrophils disrupts this regulatory axis and effectively restores antitumor immunity (81).

#### 3.2.6 Updates in mast cell

Tumor-associated mast cells resist ferroptosis themselves, and their survival along with CXCL10 secretion promotes pancreatic ductal adenocarcinoma progression and immune evasion. CXCL10 promotes epithelial-mesenchymal transition and recruits CXCR3^+^ regulatory T cells (Tregs), thereby contributing to an immunosuppressive tumor microenvironment. Targeting TAMC-derived CXCL10 with sodium cromoglycate enhances the efficacy of anti-PD-1 immunotherapy and gemcitabine, offering a promising therapeutic strategy for ductal adenocarcinoma (82).

#### 3.2.7 Updates in endothelial cells

Senescent endothelial cells secrete exosomal SLC1A5, which is taken up by tumor cells and suppresses ferroptosis in tumor cells via the EGFR/SRC/YAP1/GPX4 pathway, thereby promoting gastric cancer progression. Inhibition of this pathway effectively reduces tumor growth and metastasis, offering a promising therapeutic strategy for obesity-driven gastric cancer (83).

#### 3.2.8 Updates in tumor microbiota

Sex-specific microbiota shapes immune responses in cancer. In bladder cancer, Alistipes shahii in females produces lurasidone, which induces ferroptosis in RETNLG^+^LCN2^+^ neutrophils and enhances antitumor immunity. In contrast, males lack this advantage, resulting in increased immunosuppression (84). In colorectal cancer, curcumin modulates the gut microbiota to increase CD8^+^ T cell infiltration and ferroptosis, thereby reducing tumor growth (85). Recent advances in the tumor microenvironment are summarized in **Table 1**.

**Table 1:** Relevant progress of tumor microenvironment.

No.	Reference	Research focus	Role in TME	Mechanism/signaling pathway	Key findings	Clinical significance
(71)	Zhou L, et al., Nat Cancer, 2025	GPX4 palmitoylation regulates ferroptosis sensitivity.	Inhibits CD8^+^ T cell infiltration.	ZDHHC8 promotes GPX4 membrane localization and stability; PF-670462 degrades ZDHHC8, reducing GPX4 palmitoylation.	GPX4 palmitoylation is essential for anti-ferroptotic function.	PF-670462 combined with PD-1 blockade enhances tumor suppression.
(72)	Wang J, et al., J Exp Clin Cancer Res, 2025	BIN1 regulates ferroptosis and immune microenvironment in NSCLC.	Inhibits CD8^+^ T cell infiltration.	BIN1 affects ferroptosis via BIN1/G3BP1/STAT1/GSH pathway and T cells via CXCL10/CCL5.	BIN1 deficiency weakens immune cell function and promotes tumor survival.	Targeting BIN1/G3BP1/STAT1 can enhance ferroptosis and immune therapy.
(73)	Ma M, et al., Cancer Lett, 2025	BCL-2 inhibitor with radiotherapy induces ferroptosis.	Induces immunogenic ferroptosis.	GPX4 ubiquitination activates ferroptosis, cGAS-STING–IFN-I signaling, and CD8^+^ T cell activation.	Ferroptosis enhances immune clearance of tumors.	GPX4 degradation offers a ferroptosis-based treatment window.
(74)	Xiang B, et al., Nat Immunol, 2025	PCIF1 regulates ferroptosis sensitivity and CD8^+^ T cell immunity.	PCIF1 knockout enhances immune activity and reduces ferroptosis sensitivity	PCIF1 inhibits ferroptosis suppressor genes (e.g., Fth1, Slc3a2), reducing CD8+ T cell activation.	PCIF1 knockout improves CD8^+^ T cell activity and response to immunotherapy.	PCIF1 is a potential target to enhance CD8^+^ T cell function.
(75)	Zhao, et al., Immunity, 2025	CD8^+^ T cell ferroptosis in SCD.	Promotes T cell dysfunction and immune evasion.	SCD disrupts genome architecture, reducing SLC7A11 and H_2_S levels, inducing CD8^+^ T cell ferroptosis.	Restoring H_2_S or enhancing SLC7A11 restores CD8^+^ T cell immunity.	Provides a strategy for tumor prevention in SCD patients.
(76)	Li D, et al., Biomark Res, 2025	ROR1 CAR-T cells with ferroptosis inducers.	Enhances ferroptosis sensitivity in NSCLC.	ROR1 CAR-T cells activate ACSL4 and lipid peroxidation, inducing ferroptosis combined with RSL3.	Improves ferroptosis sensitivity in NSCLC cells.	Suggests a novel combination therapy for NSCLC.
(77)	Wan J, et al., Nat Cell Biol, 2025	LDHB-induced CD8^+^ T cell dysfunction via disulfidptosis.	Promotes immune evasion and tumor progression.	LDHB depletes NADPH and induces disulfidptosis, leading to CD8^+^ T cell exhaustion.	Targeting LDHB restores CD8^+^ T cell function and antitumor immunity.	Improves efficacy of PD-1 inhibitors in solid tumors.
(78)	Chen L, et al., Adv Sci, 2025	Macrophage ferroptosis in AAA progression.	Promotes inflammatory TME and AAA progression.	SENP3 de-SUMOylates CTH, suppressing H_2_S production and inducing macrophage ferroptosis.	SENP3–CTH–H_2_S axis is critical for AAA development.	H_2_S donors like ATB346 show therapeutic potential.
(79)	Ju Y, et al., Gut Microbes, 2025	TAM ferroptosis and colorectal cancer.	Promotes inflammation and tumor progression.	Gsta4 prevents macrophage ferroptosis and maintains pro-tumor bystander effects.	Gsta4 loss induces ferroptosis and blocks pro-tumor signaling.	Targeting Gsta4 can inhibit microbiota-driven tumor progression.
(80)	Tariq HK, et al., Cell, 2025	MDSC ferroptosis regulation by TIPE2.	Enhances MDSC immunosuppressive function.	TIPE2 promotes PUFA-PE/PC accumulation, upregulating ACSL4 and downregulating GPX4.	TIPE2 maintains MDSC-mediated immunosuppression.	TIPE2 inhibition enhances ferroptosis-inducer and PD-L1 inhibitor efficacy.
(81)	Zeng W, et al., Cancer Res, 2025	Ferroptosis in neutrophils and breast cancer chemoresistance.	Suppresses CD8^+^ T cell activity.	Fer-CD4^+^ T cells regulate neutrophil ferroptosis via IL1β/IL1R1/NF-κB signaling.	Ferroptotic neutrophils release PGE2 and IDO, suppressing CD8^+^ T cells.	Targeting Fer-CD4^+^ T cells or IL1R1^+^ neutrophils restores antitumor immunity.
(82)	Yin H, et al., Adv Sci, 2025	TAMCs and ferroptosis in PDAC.	Promotes tumor progression and immune evasion.	TAMCs resist ferroptosis and secrete CXCL10, recruiting CXCR3^+^ Tregs and promoting EMT.	TAMCs play a key role in PDAC immune evasion.	Targeting TAMCs or CXCL10 improves PDAC response to immunotherapy and chemotherapy.
(83)	Zhang Y, et al., Free Radic Biol Med, 2025	Senescent endothelial cells and ferroptosis in gastric cancer.	Promotes tumor proliferation and invasion.	Exosomal SLC1A5 suppresses ferroptosis via EGFR/SRC/YAP1/GPX4 signaling.	SLC1A5-mediated ferroptosis inhibition drives gastric cancer progression.	Targeting SLC1A5 restores ferroptosis sensitivity in gastric cancer.
(84)	Zhu Q, et al., Nat Immunol, 2025	Gut microbiota and ferroptosis in bladder cancer.	Drives sex-specific immune responses.	Akkermansia shahii produces lurasidone, inducing ferroptosis in neutrophils.	Ferroptosis regulates sex-specific immune microenvironment changes.	A. shahii or its metabolites have potential as immune enhancers.
(85)	Zhou H, et al., Food Funct, 2025	Curcumin and ferroptosis in colorectal cancer.	Enhances CD8^+^ T cell infiltration and ferroptosis.	Curcumin remodels microbiota, activating the CXCL10–CXCR3 axis and inducing ferroptosis.	Curcumin enhances the “immune activation–ferroptosis” pathway in colorectal cancer.	Holds potential as an adjunct therapy in colorectal cancer.

## 4 Conclusion and perspectives

Recent advancements in molecular mechanisms of ferroptosis primarily focus on three key areas. (1) Lipid peroxidation plays a crucial role in regulating ferroptosis sensitivity, with enzymes such as ACSL4 not only promoting tumor metastasis but also enhancing ferroptosis susceptibility. Additionally, molecules like FABP7 suppress immune cell-induced ferroptosis by modulating lipid metabolism, while SCARB1 inhibits ferroptosis through cholesterol and coenzyme Q10 regulation. Non-coding RNAs, such as lncRNA-RRFERV and CRCMSL, further influence ferroptosis by altering lipid profiles. (2) Iron metabolism is another critical aspect, and disruptions in iron homeostasis strongly affect ferroptosis. Lysosomal iron activation and ferritinophagy have been identified as key mechanisms, while regulators like NCOA4 and CPEB4 modulate iron levels and ferroptosis sensitivity. Mechanical stress and epigenetic factors, such as LTFe-enhanced iron transport, also play significant roles. (3) The redox system remains a central focus, with GPX4 stability being tightly controlled by post-translational modifications such as palmitoylation, methylation, and hydroxylation, which affect its antioxidant function. Mechanisms like GPX4-targeted degradation and alternative antioxidant pathways, including SIRT2-regulated GCLC desuccinylation, highlight cancer cells’ resistance to ferroptosis. Together, these findings underscore the pivotal roles of lipid peroxidation, iron metabolism, and redox systems in ferroptosis regulation.

Advancements in the TME focus on several key aspects. Ferroptosis in tumor cells plays a dual role in immune regulation, as DAMPs released by ferroptotic cells can activate antitumor immune responses but may also promote an immunosuppressive TME mediated by polarizing M2 macrophages and suppressing T cell functions. Immune cells regulate ferroptosis in a bidirectional manner, with CD8^+^ T cells and CAR-NK cells promoting ferroptosis through IFN-γ secretion, while tumor-associated macrophages and adipocytes inhibit ferroptosis via TGF-β1 and miR-660-5p. Ferroptosis impacts immune cell function, as it can impair the tumoricidal activity of CD8^+^ T cells, NK cells, and dendritic cells, while promoting the immunosuppressive M2 phenotype in macrophages. Additionally, ferroptosis in regulatory T cells (Tregs) reduces immunosuppression but risks autoimmunity. Cell-type-specific ferroptosis mechanisms also reshape the TME. For instance, ferroptotic neutrophils release immunosuppressive factors like prostaglandin E2, fostering chemoresistance in breast cancer, while tumor-associated mast cells resist ferroptosis and secrete CXCL10, promoting immune evasion in pancreatic cancer. Similarly, endothelial cells and tumor microbiota influence ferroptosis through pathways that modulate tumor growth and immune responses. These findings highlight ferroptosis as a key modulator of TME dynamics, offering novel therapeutic opportunities.

Based on advancements in ferroptosis mechanisms and its interplay with the TME, several promising therapeutic strategies emerge. Targeting ACSL4 is particularly effective for metastatic tumors, as ACSL4 increases membrane fluidity, promoting metastasis but also heightening ferroptosis sensitivity. Therapies targeting ACSL4, combined with ferroptosis inducers, could selectively eliminate metastatic cancer cells, especially in ovarian and breast cancers. Co-targeting GPX4 and redox systems offers another promising approach. GPX4 stabilization via palmitoylation or methylation confers resistance to ferroptosis, but therapies like GPX4-AUTAC or inhibitors of its stabilization, combined with oxidative stress-inducing agents, could enhance ferroptosis-mediated tumor killing, particularly in triple-negative breast cancer. Additionally, combining ferroptosis inducers with immunotherapy, such as anti-PD-1 antibodies, can amplify antitumor immunity by inducing ferroptosis-dependent DAMP release, activating dendritic cells and CD8^+^ T cells while reducing immunosuppressive macrophages and Tregs. This combination therapy shows strong potential in cancers like lung cancer and melanoma. Together, these strategies leverage ferroptosis-related vulnerabilities and TME dynamics, offering novel and precise avenues for cancer treatment.

Another dilemma with ferroptosis therapy comes from the use of inducers. While ferroptosis inducers, such as Erastin and RSL3, have shown promising antitumor effects, their potential off-target toxicity in healthy cells remains a significant concern. Strategies to enhance tumor selectivity, such as nanoparticle delivery systems or tumor-specific activators, need further refinement. Tumor cells exhibit adaptive mechanisms, such as upregulation of GPX4 or System Xc-, to evade ferroptosis. The oxidative stress and metabolic dependencies of ferroptosis inducers are influenced by the tumor microenvironment. For instance, the presence of stromal cells or immune cells can modulate the efficacy of ferroptosis-based therapies, complicating treatment outcomes. Many ferroptosis inducers, especially small molecules, face challenges in achieving effective concentrations at the tumor site without systemic toxicity. Nanoparticles and other delivery vectors show potential, but their clinical application requires further development (86).
